# Negative and positive allosteric modulators of the α7 nicotinic acetylcholine receptor regulates the ability of adolescent binge alcohol exposure to enhance adult alcohol consumption

**DOI:** 10.3389/fnbeh.2022.954319

**Published:** 2023-04-04

**Authors:** Zachary A. Rodd, H. Scott Swartzwelder, R. Aaron Waeiss, Serhii O. Soloviov, Debomoy K. Lahiri, Eric A. Engleman, William A. Truitt, Richard L. Bell, Sheketha R. Hauser

**Affiliations:** ^1^Department of Psychiatry, Indiana University School of Medicine, Indianapolis, IN, United States; ^2^Stark Neurosciences Research Institute, Indiana University School of Medicine, Indianapolis, IN, United States; ^3^Department of Psychiatry and Behavioral Sciences, Duke University Medical Center, Durham, NC, United States; ^4^Department of Pharmacy, Shupyk National Healthcare University of Ukraine, Kyiv, Ukraine; ^5^Department of Industrial Biotechnology and Biopharmacy, National Technical University of Ukraine “Igor Sikorsky Kyiv Polytechnic Institute”, Kyiv, Ukraine; ^6^Department of Psychiatry, Laboratory of Molecular Neurogenetics, Indiana University School of Medicine, Indianapolis, IN, United States; ^7^Indiana Alzheimer Disease Research Center, Indiana University School of Medicine, Indianapolis, IN, United States; ^8^Department of Medical and Molecular Genetics, Indiana University School of Medicine, Indianapolis, IN, United States; ^9^Department of Anatomy, Cell Biology & Physiology, Indiana University School of Medicine, Indianapolis, IN, United States

**Keywords:** alpha7 acetylcholine receptor, dopamine, alcohol, adolescence, prevention

## Abstract

**Rationale and Objectives:** Ethanol acts directly on the α7 Nicotinic acetylcholine receptor (α7). Adolescent-binge alcohol exposure (ABAE) produces deleterious consequences during adulthood, and data indicate that the α7 receptor regulates these damaging events. Administration of an α7 Negative Allosteric Modulator (NAM) or the cholinesterase inhibitor galantamine can prophylactically prevent adult consequences of ABAE. The goals of the experiments were to determine the effects of co-administration of ethanol and a α7 agonist in the mesolimbic dopamine system and to determine if administration of an α7 NAM or positive allosteric modulator (PAM) modulates the enhancement of adult alcohol drinking produced by ABAE.

**Methods:** In adult rats, ethanol and the α7 agonist AR-R17779 (AR) were microinjected into the posterior ventral tegmental area (VTA), and dopamine levels were measured in the nucleus accumbens shell (AcbSh). In adolescence, rats were treated with the α7 NAM SB-277011-A (SB) or PNU-120596 (PAM) 2 h before administration of EtOH (ABAE). Ethanol consumption (acquisition, maintenance, and relapse) during adulthood was characterized.

**Results:** Ethanol and AR co-administered into the posterior VTA stimulated dopamine release in the AcbSh in a synergistic manner. The increase in alcohol consumption during the acquisition and relapse drinking during adulthood following ABAE was prevented by administration of SB, or enhanced by administration of PNU, prior to EtOH exposure during adolescence.

**Discussion:** Ethanol acts on the α7 receptor, and the α7 receptor regulates the critical effects of ethanol in the brain. The data replicate the findings that cholinergic agents (α7 NAMs) can act prophylactically to reduce the alterations in adult alcohol consumption following ABAE.

**Graphical Abstract:**
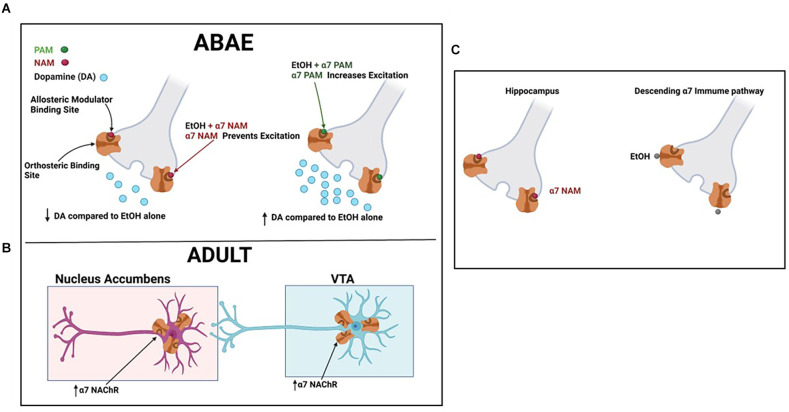
Created with BioRender.com.

## Introduction

One could argue that the largest, most continuous voluntary neurodevelopment experiment has been humankind’s alcohol consumption during adolescence. Throughout the world, multiple intervention programs have been conducted to reduce adolescent alcohol consumption, but the data indicate that the rate of adolescent alcohol consumption has remained a major public health concern (Patrick et al., 2017; Substance Abuse and Mental Health Services Administration (SAMHSA), 2018; Eisenberg et al., 2022; Kreski et al., 2022; Lines et al., 2022). The major epidemiological alteration in adolescent alcohol consumption is the increase in the overall rate of binge drinking [i.e., 4+/5+ drink/occasion (National Institute on Alcohol Abuse and Alcoholism (NIAAA), 2004)] during the transition from late adolescent/young adulthood into adulthood (Bell et al., 2014; Jager et al., 2015). The increased rate of binge drinking in adolescents/young adults led to the need to characterize high-intensity and extreme-intensity drinking in adolescents (Jager et al., 2015; Patrick and Terry-McElrath, 2017; Aiken et al., 2022). During the transition window from adolescence to young adult (21–26), 30% of US residents report recent bouts of binge drinking (Patrick et al., 2017; Kreski et al., 2022; Lines et al., 2022), while 11% report high-intensity drinking and 5% report extreme-intensity drinking (Patrick et al., 2017; Keyes et al., 2022).

The deleterious effects of adolescent/young adult alcohol binge drinking are numerous, diverse, and not limited to altering alcohol-related behaviors. Traditionally, the alcohol field has focused upon the well-replicated finding that adolescent alcohol consumption enhances adult alcohol consumption and the rate of adult alcohol use disorder (Chou and Pickering, 1992; Hingson et al., 2008). Specifically, epidemiological analyses have indicated that the risk of developing alcohol use disorder (AUD) is increased 1.3–1.6 times in individuals who initiate alcohol consumption before the age of 15 (Dawson et al., 2008). The effects of adolescent-binge alcohol exposure (ABAE) is not specific to alcohol consumption behaviors. Excessive adolescent alcohol consumption is associated with higher risk of developing mood disorders (depression and anxiety disorders), neurodegenerative diseases [e.g., Alzheimer’s disease (AD), Parkinson’s disorder (PD), other dementia-related Illnesses], other mental health disorders (schizophrenia), and auto-immune diseases during adulthood (Harwood et al., 2010; Langballe et al., 2015; Schwarzinger et al., 2018; Coleman et al., 2019; Barnett et al., 2022; Tucker et al., 2022). Therefore, developing interventions that prevent ABAE will benefit society by reducing adult rates of AUD and reducing the risk factors of several other disorders.

Neurologically, ABAE disrupts the normal remodeling of cortical and limbic regions that occurs during adolescence (Geidd, 2004). Replicable effects of adolescent alcohol consumption on the adult brain (Hauser et al., 2021a, b; Rahman and Bell, 2021) include alterations in the dopaminergic (hyper-dopaminergic) and cholinergic (reduction in ChAT, increase expression of the *Chrna7* in multiple brain regions), persistent modification of epigenetic factors, and alterations in the neuro- and peripheral immune systems (Spear and Swartzwelder, 2014; Mulholland et al., 2018; Hauser et al., 2019a, 2021a, b; Swartzwelder et al., 2019). The “time window” of ABAE is being worked out, and the present work is significant as it might extend several studies that suggest the effects of early-life exposures to environmental and psycho-social factors on later-life development of cognitive and neurological disorders, e.g., AD (Maloney and Lahiri, 2016). The initial perturbation is maintained and later further triggered through epigenetic mechanisms, i.e., the Latent Early-life Associated Regulation (LEARn) pathway, derived from studies in rodents and primates (Lahiri et al., 2009; Wu et al., 2011).

The ABAE-induced adult hyper-dopaminergic system has been observed in multiple studies using distinct methodological approaches. Adolescent ethanol consumption or peripheral administration of ethanol increases basal dopamine levels and/or dopamine reuptake in the AcbSh during adulthood (Sahr et al., 2004). In contrast, comparable adult EtOH exposure does not produce similar effects (Pascual et al., 2007, 2009). Similarly, adolescent voluntary EtOH consumption resulted in a hyperdopaminergic response to nicotine during adulthood which was not observed following comparable adult EtOH consumption (Waeiss et al., 2019). In Wistar and alcohol-preferring (P) rats, ABAE results in a leftward and upward shift of the dose-response curve (increased sensitivity) for EtOH to stimulate dopamine release in the AcbSh after posterior ventral tegmental area (VTA) microinjection (Hauser et al., 2021b). ABAE-induced hyper-dopaminergic system during adulthood is thought to be part of the biological basis for the enhanced adult EtOH consumption observed following ABAE. Activation of the mesolimbic dopamine pathway is considered critical for the development of alcohol consumption and the progression to AUD (Rodd et al., 2004).

Exposure to a variety of drugs of abuse (alcohol, nicotine, opioid, and cocaine) during adolescence results in reduced ChAT expression during adulthood (Wilson et al., 1994; Abreu-Villaça et al., 2004). Recent data examining compensatory alterations in nicotinic acetylcholine receptors (NAChR) in response to the ABAE-induced reduction in ChAT has indicated an increased expression of *Chrna7* (and other NAChRs) in the posterior VTA and AcbSh (Hauser et al., 2019a, 2021b). Voluntary adolescent EtOH consumption in P rats increases by 2-fold the number of homomeric α7-immunoreactive (IR) NAChR neurons in the posterior VTA and increases the protein expression of the α7 receptors in the posterior VTA (Waeiss et al., 2019). Convergently, ABAE induces a significant increase in the *Chrna7* (α7) gene expression in the posterior VTA and the AcbSh (Hauser et al., 2019a, 2021b). In addition, adolescent intermittent ethanol caused a persistent increase in adult histone methylation at histone 3 lysine 9 dimethylation (H3K9me2) near the NTRK1 neurotrophic receptor tyrosine kinase 1 (NTRK1) gene and DNA methylation in promoter regions of ChAT, both of which were remediated by wheel running (Vetreno et al., 2020).

Research characterizing the α7 receptor has reported unique properties of the receptor. In humans, a chimeric gene (*CHRFAM7A*) that encodes a protein that acts as an innate α7 NAM (Araud et al., 2011). Polymorphisms of the *CHRFAM7A* gene (reduction in function) results in increase susceptibility to several diseases (e.g., schizophrenia; Akbarian and Kundakovic, 2015) observed following ABAE. Conversely, over-expression of the α7 is associated with a number of neuropsychiatric disorders. An increase in the expression of the α7 (approximately 30%, comparable to what is observed preclinically following ABAE treatment in rats) results in alterations in the expression of epigenetic factors and other genes, differential regulation of developmental signaling, and altered neurogenesis and synaptogenesis (c.f., Meganathan et al., 2021).

The α7 receptor is associated with non-canonical activation of neurons, immune, and other cells. On leukocytic T cells, the α7 receptor increases Ca^2+^ signaling through activation of a protein complex and TCR/CD3 (increasing protein tyrosine kinase; Razani-Boroujerdi et al., 2007). Phosphocholine-induced inhibition of IL-1b release through metabotropic signaling and protein coupling through the α7 (Richter et al., 2018). In the ventrohippocampal-striatal synapse, activation of the α7 results in sustained increase in glutamate release (Zhong et al., 2008). This α7 mediated effect is considered a main driver of facilitation of glutamatergic synaptic transmission which would increase GABA, acetylcholine, and dopamine release (Zhong et al., 2013). This α7 enhancement of glutamate levels could be the biological basis for the long-term enhancement of glutamate levels in the AcbSh produced by chronic EtOH + nicotine co-use (Deehan et al., 2015). In contrast to the canonical belief that a receptor is either a ligand-gated ion channel or a G-coupled receptor, the α7 is a dual ionotropic and metabotropic receptor. Specifically, activation of the α7 NAChR increases Ca^2+^ influx (ligand-gated ion channel) and/or increases Gαq (sustained release of Ca^2+^) in the neuron (Kabbani and Nichols, 2018). In addition, α7 desensitization period is short and the recovery period is fast, which makes the α7 very different from other NAChRs (Papke et al., 2009). The properties of the α7 NAChR and the published data indicating the importance of this receptor in mediating the effects of ABAE-induced adult consequences indicate that the α7 NAChR is a valid candidate to develop pharmacotherapeutics to counter ABAE (Rodd et al., 2020).

EtOH directly acts on the α7 receptor. Oocyte determination of EtOH actions at the α7 receptor indicated dual excitatory and inhibitory responses (Doyon et al., 2013a). Similar results were determined using *in vitro* electrophysiological assessment of the action of EtOH on the α7 receptor (Doyon et al., 2013b). However, the complexity of the α7 receptor reduces the confidence in *in vitro* analysis of the effects of EtOH on the receptor (e.g., G-couple protein properties vs. ligand gated ion channels and lack of functional inputs onto selected, stabilized, dissociated neurons). The initial experiment provided the needed data that would examine the *in vivo* effect of activating the α7 receptor on the ability of EtOH to stimulate posterior VTA dopamine neurons. An increase in the activation of the mesolimbic dopamine system suggests the animal is experiencing reinforcing stimulation that could indicate an increased propensity to perform behaviors to obtaining this effect (e.g., alcohol consumption; Rodd et al., 2004).

The effects of adolescent alcohol consumption on adult alcohol consumption can be characterized as a “division of thirds”. One third of American adolescents do not drink alcohol while another third consumes alcohol during adolescence but does not develop into adult AUD. The final third are individuals that consume alcohol (binge drinking) during adolescence and are diagnosed with AUD during adolescence or adulthood (Tripodi et al., 2010). There are many types of clinical interventions to treat adolescent alcohol consumption. Cochrane reviews have indicated that the clinical interventions have “poor” outcomes (Foxcroft and Tsertsvadze, 2011; Carney et al., 2016).

Recent preclinical data have indicated possible prophylactic pharmacological intervention to prevent the deleterious consequences of ABAE on adult alcohol consumption and biological factors. Pretreatment with the α7 negative allosteric modulator (NAM) dehydronorketamine (DHNK) before ABAE prevented the enhancement of alcohol consumption detected during the acquisition of EtOH consumption during adulthood and EtOH relapse drinking produced by ABAE (Rodd et al., 2020). In addition, administration of the α7 receptor agonist AR-R17779 during adolescence mirrored the effects of ABAE in adult ethanol consumption by increasing acquisition and relapse drinking (Rodd et al., 2020). However, this does not indicate that activation of the α7 allosteric binding site mediates the effects of ABAE. Parallel research has indicated that pretreatment with galantamine (a cholinesterase inhibitor) blocked ABAE-induced increases in the expression of genes associated with the innate immune system (*TLR4* and *pNF-κB*) and histones/chromatin related genes (*RGE* and *HMGB1*) and during adulthood (Crews et al., 2021). Preclinical evidence from independent laboratories has indicated that pharmacological intervention can act prophylactically to prevent the deleterious effects of ABAE on adult neurophysiology and behavior. The reported data examined the efficacy of SB-277011-A (an α7 NAM and a D3 antagonist; Zheng et al., 2016) to act prophylactically to prevent ABAE-induced enhancement of adult alcohol consumption.

The allosteric binding site on the α7 receptor could have bi-directional effects on ABAE-induced enhancement of adult alcohol consumption. To date, the parametrics of ABAE (amount of EtOH required to produce the adolescence consequences, length and duration of ABAE, and more) have not been established. A α7 positive allosteric modulator (PAM) could amplify the effects of EtOH during adolescence exposure to produce enhancement of adult alcohol consumption (possible synergistic effects of low-dose EtOH exposure). The current research project also examined the effects of co-administration of the α7 PAM PNU with subthreshold (2.0 g/kg) adolescent EtOH exposure on adult alcohol consumption in male and female Wistar rats.

## Methods

### Subjects

Wistar rats are maintained at Indiana University School of Medicine as a single-generation colony (IUSM; Indianapolis, IN). The Taconic P (tP) rat is a substrain of the P rat that was maintained at Taconic Farms for over 10 years before being returned to Indiana University (2006). Similar to the Wistar colony, the tP colony complete life cycle (breeding/rearing) was conducted in the same building as the research was performed. Same building care eliminates the stress from shipping adolescents.

Animals care facilities at IUSM are fully accredited by the Association for the Assessment and Accreditation of Laboratory Animal Care. Research performed in the current experiments were approved by the IUSM Institutional Animal Care and Use Committee (IUSM IACUC) and were in accordance with the guidelines of the Institutional Care and Use Committee of the National Institute on Drug Abuse, the NIH, and the Guide for the Care and Use of Laboratory Animals (2011).

### Co-Administration of EtOH and the α7 receptor agonist AR-R17779 into the posterior VTA on extracellular dopamine levels in the AcbSh

#### Microinjection-microdialysis protocol

**Figure 1:**
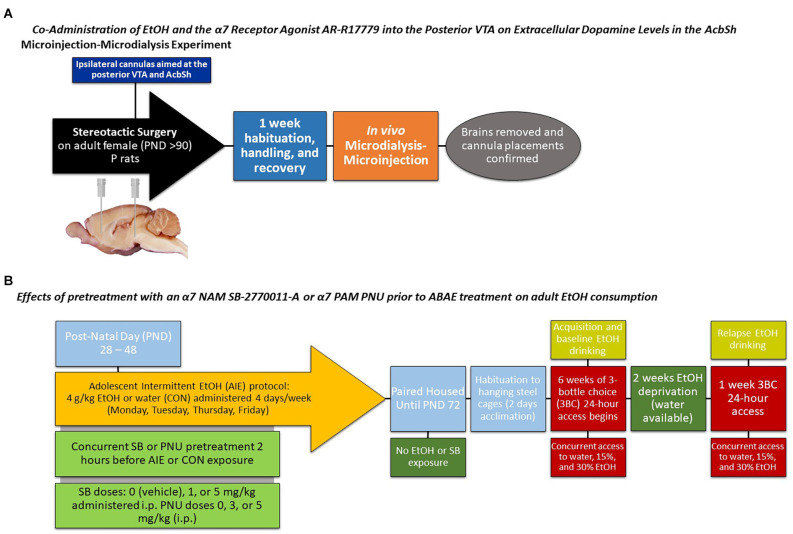
Depicts a timeline for the **(A)** microinjection-microdialysis experiments and **(B)** adult alcohol intake experiments.

Detailed methodology for the microinjection-microdialysis procedure has been published elsewhere (Figure 1; Deehan et al., 2015; Waeiss et al., 2019, 2020; Hauser et al., 2021b). Surgery was performed in adult rats (>PND 90). Rats were ipsilaterally implanted with guide cannulas aimed at the posterior VTA (AP −5.6 mm, ML +2.1 mm, DV −8.0 mm) and AcbSh (AP +1.5 mm, ML +2.0 mm, DV −5.3 mm). Surgical details are described previously (Waeiss et al., 2019). Because of the delicate nature of the experiment (rats moving their heads can destroy equipment or HPLC assemblies), rats were handled daily after surgery until microdialysis testing. The researchers believe that frequently handling of rats is beneficial to the rats and reduces stress associated with experiments (grasping of rats to insert microinjections, etc.).

Microdialysis analyses of DA levels in the AcbSh were performed using loop-style probes that are manufactured in the laboratory (Waeiss et al., 2020; Hauser et al., 2021b). The production of probes has been described in detail in previous publications (Waeiss et al., 2020; Hauser et al., 2021b). Under isoflurane anesthesia, microdialysis probes were inserted (3.0 mm below the guide cannula) into the AcbSh 24-h before the performance of the microinjection-microdialysis experiment.

During the microinjection-microdialysis experiments, rats are awake and are active in the testing chambers (freely moving). Subjects are connected to the microdialysis apparatus (detailed in Hauser et al., 2021b) and a 90-min washout period (removes residue from probe and stabilizes all neurotransmitter levels) was performed. Samples were collected in 20-min intervals during the five baseline samples and following microinjections into the posterior VTA.

Experimenter controlled microinjections were carried out with an electrolytic microinfusion transducer (EMIT) system (Waeiss et al., 2019; Hauser et al., 2021b). Microinjection of test solutions into the posterior VTA followed a cycle of 5-s microinjection and 15-s timeout period. Microinjections occurred over a 10-min period (30 injections in total, 3 μl total injection volume).

All microdialysis samples were collected and stored for testing using established methods (Waeiss et al., 2019). Samples were analyzed for dopamine content through the use of an high performance liquid chromatography (HPLC) system (Hauser et al., 2021b).

#### Concentrations of EtOH and AR-R17779

Adult Wistar male rats were microinjected with artificial cerebrospinal fluid (aCSF), EtOH alone (100 or 150 mg%), AR alone (500 nM) or EtOH + AR (50 mg% EtOH + 500 nM AR or 100 mg% EtOH + 500 nM AR). The total number of animals used in the experiment was 37 (*n* = 7–9/group). Past research has indicated no difference between male and female rats for the ability of EtOH microinjected into the posterior VTA to stimulate dopamine release in the AcbSh (Hauser et al., 2021b). Female rats were not included in this study because of financial limitations and the low likelihood to observe sex differences.

#### Histological verification

At the end of the schedule experiment, animals were sacrificed and a solution of 1% bromophenol blue dye was injected into the posterior VTA and AcbSh. Following storage (−80°C), brains were sectioned (40-μm) and placed on slides. Staining with cresyl violet allowed for delineation of brain regions. Site verification was confirmed using the atlas of Paxinos and Watson.

### Pretreatment with an α7 NAM SB-2770011-A prior to ABAE treatment on adult EtOH consumption

The method of testing the effects of ABAE on adult alcohol consumption has been described previously (Rodd et al., 2020; Figure 1). In the current experiment, there were six treatment groups. Adolescent male (*n* = 35) tP rats were used in the experiment. Visual representation of the experimental design has been published (Figure 1). ABAE treatment began on PND28, were intermittent (4 days of the week), and EtOH treatment (4.0 g/kg/day; 25% v/v EtOH) was performed through gavage. Other rats received comparable gavage treatment (equivalent volume) of water (CON rats) on the same days and for the same duration of treatments. ABAE and CON rats were randomly assigned to one of three doses (0, 1, or 5 mg/kg) of SB-277011-A (SB; Sigma-Aldrich, Inc, St. Louis, MO, USA) that was administered 2 h prior to ABAE or CON treatment (PND 28–48; a total of 14 treatments). Rats were not exposed to SB at any other time. Rats were group housed until the testing of adult alcohol consumption. Previous research has indicated no six differences in the ability of a α7 NAM to block/prevent the effects of ABAE on adult alcohol consumption (Rodd et al., 2020). Because of financial limitations on research and the low probability to observe a sex difference, females were excluded from the study.

#### Adult EtOH consumption

To remove any potential bias, collection of data (fluid intake) was conducted blind to adolescent treatment conditions.

For the most accurate assessment of adult alcohol intake, all rats were placed into individual hanging steel cages 2 days prior to the initial exposure to adult EtOH (PND 73). The hanging steel cages are equipped with features that reduce stress to the rats (unanchored perch in each cage). The rats use the perch to rest, avoid standing on the wire mesh, and to sleep. Adding a perch to hanging steel cages is required by the IUSM IACUC.

Throughout the experiment, food and water was constantly available to the rats (*ad libitum*). EtOH solutions made available on PND 75 (15 and 30% EtOH v/v) were created from a stock of ethyl alcohol (190 proof; McCormick Distilling Co., Weston, MO, USA).

The research protocol has been employed multiple times in the past to examine acquisition and relapse EtOH consumption in rats (Rodd et al., 2020). Briefly, rats are given free-choice access to water, 15 and 30% EtOH (3 bottle-choice) for 6 weeks (measurements taken daily). EtOH solutions (but not water) are removed for 2 weeks (deprivation/abstinence). EtOH solutions were returned for a 2-week re-exposure period. This protocol is well-established to produce an increase in EtOH consumption upon re-exposure to EtOH (alcohol deprivation effect, ADE; Rodd et al., 2004).

### Effects of co-administration of the α7 PAM PNU simultaneously with low-dose ABAE treatment on adult EtOH consumption

Adolescent male (*n* = 39) and female (*n* = 41) Wistar rats were used in the experiment. The experimental design was a 2 × 3 between subject protocol. Rats received low-dose ABAE treatment (2 g/kg, gavage) or water which began on PND28 and were simultaneously administered PNU-120596 (0, 3, or 5 mg/kg; Sigma-Aldrich, Inc, St. Louis, MO, USA). ABAE treatment was intermittent (4 days of the week) until PND48. ABAE exposed to 2 g/kg EtOH should have resulted in a peak blood ethanol concentration (BEC) of approximately 35–40 mg% (Vetreno et al., 2020). The primary reason why both sexes were not tested in the two adult conditions was primarily financial. These prolonged experiments are extremely costly. The same real life constraints (and the general unwillingness for funding agencies to support research preventing the negative consequences of ABAE) limited the dose of EtOH exposure during adolescence and dose of PNU tested. Individuals reading scientific manuscripts must ask the following questions; could they obtain funding for the ideal research project they demand from a publication, and if they were a reviewer for a grant application would they support funding for a full parametric analysis?

### Males—24-h free-choice drinking

The goal of the experiment was to have Wistar rats consuming pharmacological relevant levels of alcohol. The most reliable manner to obtain significant BECs in Wistar rats is to provide a palatable alcohol solution (beer). McGregor and colleagues have repeatedly published that all rats will consume beer at a level that produces significant BECs (McGregor et al., 2005; Hargreaves et al., 2009a, b, 2011). In addition, adolescent beer drinking in outbred rats increases beer drinking during adulthood (Hargreaves et al., 2011). A difference between our procedure and McGregor’s (and others’) protocols is that we do not use pilsners. In a taste test of 54 microbrews, we have determined that rats (Wistar and tP, male and female) prefer India Pale Ales (IPA) or Red Ales (and dislike pilsners). Therefore, we used a West Coast IPA (7.7% alcohol) and a Toasted Red Ale (5.2% alcohol).

To remove any potential bias, collection of data (fluid intake) was conducted blind to adolescent treatment conditions. Rats were treated identical to the tP rats. Rats are given free-choice access to water, 5.2% and 7.7% EtOH (3 bottle-choice) for 6 weeks (measurements taken daily). EtOH solutions (but not water) are removed for 2 weeks (deprivation/abstinence). EtOH solutions (beer) were returned for 2-week re-exposure period. This protocol is well-established to produce an increase in EtOH consumption upon re-exposure to EtOH (alcohol deprivation effect, ADE; Rodd et al., 2004).

### Females—1-h daily operant beer self-administration

There are many behavioral measures that could be tested under operant conditions that cannot be assessed through free-choice drinking. The current experiment examined the effects of low-dose ABAE and PNU exposure on the acquisition, maintenance, extinction, seeking, and relapse behavior. Briefly, rats were placed in a standard two lever operant chamber (water or 7.7% West Coast IPA as reinforcer).

Without training, female Wistar rats were placed into the operant chambers and allowed to self-administer water or 7.7% West Coast IPA every day during a 1-h session. Detailed methodology is available in other publications (Rodd-Henricks et al., 2002a, b; Hauser et al., 2019b). The fixed ratio (FR) requirement for the beer solution was an FR1 for the initial 4 weeks, FR3 during weeks 5–6, and an FR5 during weeks 7–8 (maintained on this FR for the rest of the experiment).

Female rats were given 8 weeks of beer access. Rats were then exposed to extinction training during weeks 9 (no beer or water available in operant chambers). Rats were then maintained in their homecages for 2 weeks. Rats were then tested for context-induced beer-seeking [Pavlovian Spontaneous Recovery (PSR)] for four sessions. After seeking testing, rats were maintained in their homecages for a week. Rats were then returned to the operant chamber with beer and water available for self-admininstration.

#### Statistical analysis

The statistical analysis methodology outlined by Keppel and Zedeck (1986) was used. Briefly, mixed factor ANOVAs were conducted for the overall analysis. The between subject factors were Dose of SB and ABAE. The single within subject factor of Week and the dependent measure of weekly average intake were used. The last 3 days of EtOH consumption prior to the abstinence period was used as baseline for the relapse analyses. The overall analysis was a mixed factor ANOVA with between subject factors of SB and ABAE AEH and within subject factors of day (baseline intake compared to intake levels following EtOH re-exposure). *Post-hoc* comparisons for significant differences for between subject variables was the Tukey’s b analyses. To avoid violation of the ANOVA assumption of independent measures, proper within subject analysis (*t*-tests and orthogonal contrasts) were used. We only report the results of the *t*-test analyses. In past publications, we have detailed the correct equations of Type I error rate inflation (Rodd et al., 2020). Given the effect size of the current dataset there is little likelihood of Type 1 error rate inflation for any reported analyses (c.f., Rodger, 1967).

## Results

### The effects of co-administration of EtOH and the α7 receptor agonist AR-R17779 into the posterior VTA on extracellular dopamine levels in the AcbSh

**Figure 2:**
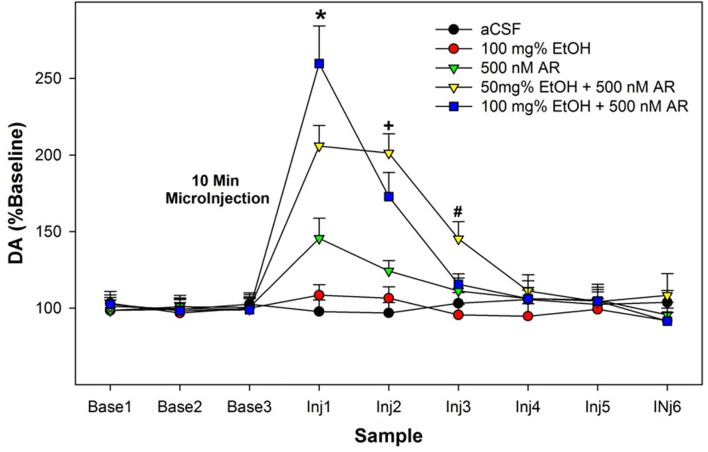
Depicts the average extracellular DA levels in the AcbSh in P rats following microinjects of EtOH, AR, or EtOH+AR into the posterior VTA. *indicates in 100 E + 500 AR >50 E + 500 AR >500 AR >aCSF and 100 E . ^+^indicates that two E + AR >500 AR >aCSF and 100 E. ^#^indicates 50 E + 500 AR >all other groups.

The overall statistical analysis performed on the dependent measure of the %change of DA levels from baseline in the AcbSh indicated that there was a synergistic effect of combining EtOH and AR (Group × Sample interaction term—*F*_42,186_ = 2.9; *p* < 0.001; Figure 2). The significant interaction term was decomposed by examining the effect of Group at each sample time (performing individual ANOVAs). There were no significant Group differences during the three baseline samples and for the 4th–6th samples obtained after the microinjection procedures (Group *F*_4,32_ values < 1.04; *p* values > 0.85). There were significant Group differences during the 1st–3rd samples obtained after microinjections of assigned compounds into the poster VTA (Group *F*_4,32_ values >13.81; *p* values < 0.002). *Post-hoc* comparisons (Tukey’s b) revealed that during the 1st post-microinjection period there were significant group differences; 100 mg% EtOH + 500 nM AR >50 mg% EtOH + 500 nM AR >500 nM AR >100 mg% EtOH and aCSF controls. Preliminary data (Waeiss et al., 2019) indicated that microinjections of 500 nM AR into the posterior VTA did not alter dopamine release in the AcbSh. We did not anticipate that microinjection of 500 nM AR would significantly increase dopamine levels in the AcbSh. During the 2nd post-microinjection period, *post-hoc* comparisons revealed significant group differences: 50 mg% EtOH + 500 nM AR and 100 mg% EtOH + 500 nM AR >aCSF, 100 mg% EtOH, and 500 nM AR. During the 3rd post-microinjection period, *post-hoc* comparisons revealed that rats administered 50 mg% EtOH + 500 nM AR has significantly higher levels of dopamine in the AcbSh than all other groups.

### Effects of pretreatment with an α7 NAM SB-2770011-A prior to ABAE treatment on adult EtOH consumption

#### Acquisition

**Figure 3:**
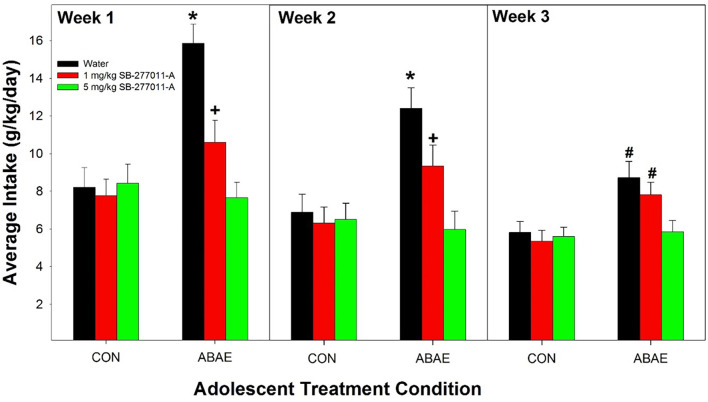
Depicts the average alcohol intake in female P rats administered the α7 NAM SB-277011-A 2 h before ABAE treatment during adolescence on the adult consumption of EtOH. *indicates EtOH consumption in water-ABAE rats >1 mg and 5 mg/kg SB rats. ^+^indicates 1 mg/kg >5 mg/kg SB rats. ^#^indicates water-ABAE and 1 mg/kg SB >5 mg/kg SB rats.

The dependent measure was the weekly average of EtOH intake. Weekly averages were used in the analysis for acquisition of drinking. If daily intake was analyzed, there is a limited time period to observe drinking behavior because of the elimination of the degrees of freedom. The overall analysis indicated that pretreatment with SB prevented the ABAE-induced increase in EtOH consumption during acquisition (Figure 3). Statistically, there was a significant ABAE × SB Dose × Week interaction term (*F*_4,147_ = 3.68; *p* < 0.001). The significant interaction 3-way interaction was decomposed by examining the effects of ABAE × SB Dose on the weekly averages. There were significant 2-way interactions (ABAE × SB Dose) for each average weekly intake values (*F*_4,147_ values > 2.37; *p* values < 0.001). Holding ABAE history constant revealed that in the CON groups, there was no effect of SB Dose (*p* values > 0.72). In ABAE rats, there was a significant effect of SB Dose (*p* values < 0.003). *Post-hoc* comparisons indicated that during the 1st and 2nd weeks of EtOH access rats given water pretreatment drank more EtOH than rats administered 1 or 5 mg/kg SB (water >1 mg/kg >5 mg/kg). During the 3rd week of EtOH access, rats pretreated with water or 1 mg/kg before ABAE consumed more EtOH during adulthood than rats pretreated with 5 mg/kg SB. To confirm that ABAE enhanced acquisition drinking during adulthood, SB dose was held constant and weekly average EtOH intake was analyzed. The analyses revealed that during the 1st–3rd weeks, the ABAE Water group consumed more EtOH than the CON Water group (*p* values < 0.0001). In rats pretreated with 1 mg/kg SB prior to adolescent treatment indicated that ABAE—1 mg/kg SB rats consumed more EtOH during the 2nd and 3rd week of acquisition compared to CON— 1 mg/kg SB rats (*p* values < 0.01). In contrast, there was no significant effect of ABAE history on adult EtOH intake (acquisition) in rats pretreated with 5 mg/kg SB (*p* values > 0.53).

#### Relapse

**Figure 4:**
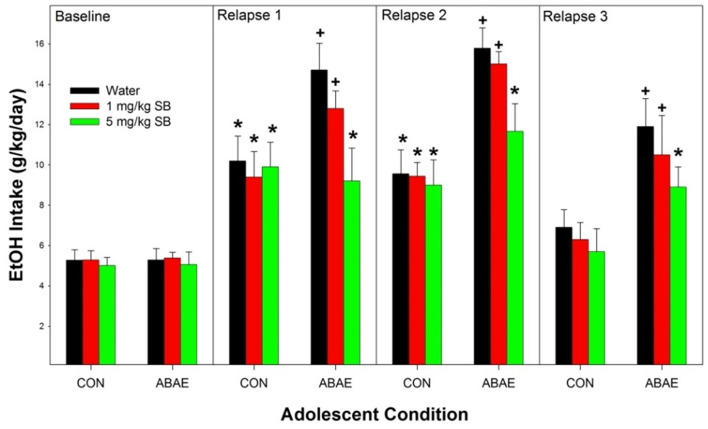
Depicts the mean (+ SEM) for female alcohol-preferring (P) rats during relapse EtOH drinking. *indicates EtOH consumption exceeds baseline intake. ^+^indicates that rat pretreated with saline prior to ABAE exposure (water and 1 mg/kg SB) consumed more alcohol than corresponding CON group.

Overall, ABAE increased relapse drinking during adulthood and pretreatment with SB suppressed ABAE-enhancement of relapse EtOH drinking. Statistically, there was a significant ABAE × SB Dose × Day interaction term (Figure 4; *F*_12,244_ = 2.3; *p* < 0.01). The 3-way interaction term was decomposed by holding the factor of “Day” constant. There was no significant effect of ABAE × SB Dose on baseline (maintenance) intake (*p* = 0.78). During the 1st–3rd relapse (ADE; individual ANOVAs performed) drinking days, there were significant ABAE × SB Dose interactions for EtOH consumed (*F*_2,32_ values > 2.24; *p* values < 0.024). The interaction term was decomposed by holding both ABAE history and SB Dose constant. During the 1st and 2nd relapse drinking day, in the CON group of rats there was no effect of SB Dose on EtOH intake (*p* = 0.0.77), but there was an effect of SB Dose in the ABAE group (*F*_2,17_ values > 11.88; *p* values < 0.01). *Post-hoc* comparisons for the ABAE group indicated that EtOH intake was significantly higher in the Water and 1 mg/kg SB group compared to the 5 mg/kg group (Tukey’s b). Examining the effects of ABAE history during the 1st relapse drinking day, there was significant CON vs. ABAE group differences for the Water and 1 mg/kg SB groups. During the 3rd relapse drinking day, the only statistical change was that in rats with a past history of ABAE the 1 mg/kg SB no longer consumed more EtOH than the 5 mg/kg SB group. To confirm that there was an increase in EtOH intake compared to baseline, within groups analyses were performed (*t*-tests; *p* < 0.05). In the CON rats, all three SB Dose groups consumed more EtOH during the 1st and 2nd relapse drinking days. In the ABAE rats, all three SB Dose groups consumed EtOH during the 1st, 2nd, and 3rd relapse drinking days.

### Effects of co-administration of the α7 PAM PNU simultaneously with low-dose ABAE treatment on adult EtOH consumption

#### Males—24-h free-choice drinking

**Figure 5:**
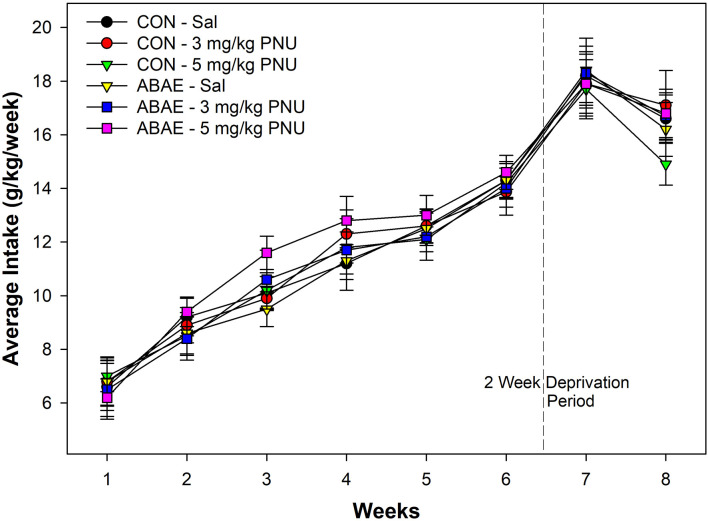
Depicts the mean (+ SEM) weekly average intake (g/kg/day) of alcohol (beer) in male Wistar rats administered a subthreshold dose of EtOH during adolescence and the α7 PAM PNU. There was a significant increase in alcohol consumption in all groups during the experiment, but not effects of ABAE or PNU treatment.

Males consumed a large amount of beer. In general, all rats consumed a large amount of beer which increased over the time of access. During the first week of access, male rats consumed on average 7.1 g/kg/day. The level of intake was significantly higher during the 6th week of access (average intake 13.8 g/kg/day. Statistically, there were no significant effects of ABAE, PNU, or ABAE × PNU interactions on beer consumption during the first 6 weeks of access (*p* values > 0.37). There was a main effect of Week (Figure 5; *F*_5,32_ = 12.6; *p* < 0.01).

There was a significant increase in beer drinking following the 2-week deprivation period (*F*_2,34_ = 10.8; *p* < 0.01). Statistically, there were no significant effects of ABAE, PNU, or ABAE × PNU interactions on beer consumption during the relapse beer drinking (*p* values > 0.46). On average, male Wistar rats consumed 125.2 g of beer per day during the first week of beer re-exposure. Given that the rats weighed on average 457.8 g during this time, rats were consuming 27.3% of their body weight in beer each day. For comparison, this would be similar to an 85 kg human consuming 23.5 kg of 7.7% beer per day.

#### Females—1-h daily operant beer self-administration

**Figure 6:**
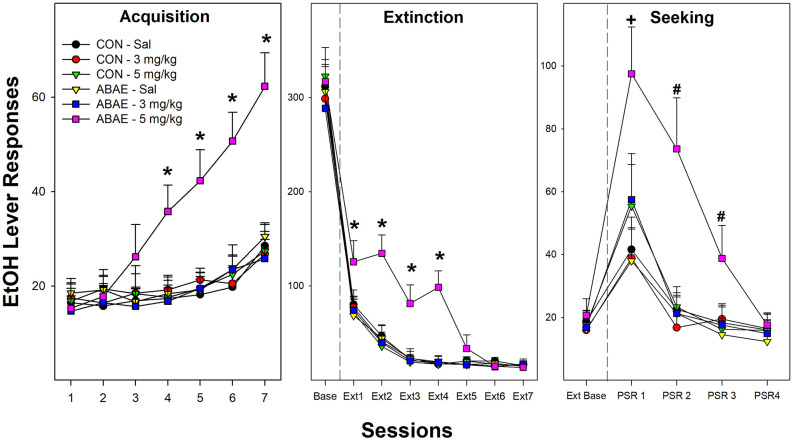
Depicts the mean (+ SEM) number of EtOH (beer) lever responses in female Wistar rats as a product of exposure to low-dose ABAE (2 g/kg) and treatment with the α7 PAM PNU. During Acquisition of responding (left panel), female rats treated with 2 g/kg ABAE and 5 mg/kg PNU during adolescence responded more on the lever associated with the delivery of beer (7.7% West Coast IPA; *indicates significant difference from all other groups). Rats treated with 2 g/kg ABAE and 5 mg/kg PNU during adolescence responded more on the lever previously associated with the delivery of beer during the first four Extinction training sessions (middle panel; *indicates significant difference from all other groups). During context-induced EtOH-seeking (right panel), female Wistar rats treated with 2 g/kg ABAE and 5 mg/kg PNU during adolescence responded more on the lever previously associated with the delivery of beer than all other groups during sessions 1–3. ^+^sign indicates that all groups displayed an increase in responding compared to extinction baseline, and 2 g/kg ABAE and 5 mg/kg PNU are significantly higher than all other groups. ^#^sign indicates that 2 g/kg ABAE and 5 mg/kg PNU are significantly higher than all other groups and higher than extinction baseline.

Examining the acquisition of beer self-administration in female Wistar rats revealed a significant Session × ABAE × PNU interaction term (Figure 6; *F*_12,368_ = 3.4; *p* < 0.01). For ease of decomposing the significant three-way interaction term, ANOVAs were performed on all groups of ABAE and PNU testing (six total). Individual ANOVAs revealed that sessions 4–13 there was a significant effect of Group (*F*_5,37_ values > 23.8; *p* values < 0.01). *Post-hoc* analyses (Tukey’s b) revealed that female rats treated with 2.0 g/kg EtOH during adolescence (ABAE) and simultaneously administered 5.0 mg/kg PNU responded more for the 7.7% beer than all other groups. After the 14th session, all groups responded equivalently. At each FR schedule of reinforcement, all rats self-administered about 60 reinforcers per session (Figure 6; middle panel). During Extinction Training, there was a Session × ABAE × PNU interaction term (*F*_12,368_ = 2.0; *p* < 0.01). ANOVAs indicated that there was a significant Group difference during Extinction Sessions 1–4 (*p* values < 0.02). *Post-hoc* comparisons (Tukey’s b) indicated that female rats treated with 2.0 g/kg EtOH during adolescence (ABAE) and simultaneously administered 5.0 mg/kg PNU responded more on the lever previously associated with the delivery of 7.7% beer than all other groups.

During context-induced alcohol-seeking testing (Figure 6; right panel), there was a significant Session × ABAE × PNU interaction term (Figure 6; *F*_8,312_ = 1.9; *p* < 0.01). The was a significant Group difference during PSR test sessions 1–3 (*F*_5,37_ values > 17.5; *p* < 0.01). Similar to acquisition and extinction training, *post-hoc* analyses revealed that female rats treated with 2.0 g/kg EtOH during adolescence (ABAE) and simultaneously administered 5.0 mg/kg PNU responded more on the lever previously associated with the delivery of 7.7% beer than all other groups. Alcohol craving is indicated in the PSR model by an increase in the level of responding during the PSR test sessions compared to responding during the last three sessions of extinction training. This within subject analysis is determined by *t*-tests. All rat groups responded more during the 1st PSR test session than during extinction (*p* values < 0.032). There were no significant effects during relapse testing.

## Discussion

The current results indicate that co-administration of EtOH enhances the activation of posterior VTA dopamine neurons (the source of increased DA in the AcbSh) by an α7 receptor agonist (Figure 2). Electrophysiological studies of dissociated neurons and oocyte research previously indicated that EtOH acts on the α7 receptor with equivocal effects (Doyon et al., 2013a). Stimulation of the α7 receptor in the posterior VTA activates DA neurons (Schilström et al., 2003). On VTA DA neurons, the D2 receptor is linked-expressed with the α7 receptor (Garzón et al., 2013). Activation of the D2 autoreceptors inhibits activation of DA neurons, while activation of the α7 receptor stimulates VTA DA neurons (Garzón et al., 2013). Classic antipsychotic drugs (inhibitors of D2 receptors) are thought to be efficacious through disinhibition of the stimulatory actions of the α7 receptor on VTA DA neurons (Garzón et al., 2013). Reduced ABAE exposure (2.0 g/kg/day) failed to alter adult EtOH consumption (Figures 5 and 6). Co-administration of the α7 PAM PNU with low-dose ABAE did result in enhanced beer self-administration in Wistar female rats (Figure 6). These data indicate that enhancing the likelihood that the α7 receptor will be activated if stimulated by another ligand (EtOH) or activator (actions of a PAM) resulted in a leftward shift in the dose response curve for EtOH ABAE consequences on adult alcohol consumption. The data generated in rats given exposure to a subthreshold dose of EtOH (2 g/kg) and the α7 PAM PNU, parallels the findings (increased acquisition, reduced rate of responding, and enhanced Alcohol-Seeking behaviors) observed in *p* and Wistar rats administered the typical (4 g/kg) ABAE (Rodd-Henricks et al., 2002a; Gass et al., 2014). These data indicate the importance of the α7 receptor in mediating the effects of EtOH in the adolescent brain.

Furthermore, reducing activation of the α7 receptor (e.g., an α7 NAM) prevents the development and expression of social stress-induced neuroadaptations (Morel et al., 2018). Similar to our findings that administration of an α7 receptor agonist during adolescence enhances adult EtOH consumption, activating the α7 receptor promotes stress-induced cellular neuroadaptations and predisposes organisms to express anxiety-like behaviors (Morel et al., 2018). Thus, ABAE-induced upregulations of the *Chrna7* gene throughout the brain (Hauser et al., 2019a, 2021b) could be a critical biological basis for the replicated finding that ABAE results in increased anxiety levels during adulthood (Sánchez-Marín et al., 2022).

The current data indicate that the co-administration of EtOH and an α7 receptor agonist into the posterior VTA synergistically increases dopamine release in the AcbSh (Figure 2). Past research has indicated that selective breeding for high alcohol preference (P, HAD-1, and HAD-2 rats) is associated with a posterior VTA–AcbSh neurocircuitry that is more sensitive (leftward and upward shift in the dose-response curve) to the stimulatory effects of nicotine in the posterior VTA (Deehan et al., 2018). The activation of the mesolimbic dopamine system is considered critical for the reinforcing properties of drugs of abuse and the regulation of alcohol consumption (Rodd et al., 2004). The hyperdopaminergic consequence of ABAE is indicated by basal neurochemical differences (Sahr et al., 2004), the increase in the genetic expression of dopamine reuptake system (Brancato et al., 2021, 2022), and the increase in sensitivity and response to stimulation during adulthood (Hauser et al., 2019a, 2021b).

EtOH and nicotine microinjected into the posterior VTA (but not nicotine or EtOH microinjected alone) results in an increase in glutamate release in the AcbSh and an increase in the expression of BDNF in the AcbSh (Waeiss et al., 2020). In addition, microinjection pretreatment of EtOH and nicotine into the posterior VTA (but not nicotine or EtOH microinjections alone) enhanced the rewarding properties of EtOH within the AcbSh. The current study suggests that the activation of the α7 receptor in conjunction with EtOH administration into the posterior VTA could be the biological basis for these observed effects (Figure 2). A limitation of the current study is that the neurochemical experiment occurred in adult and not adolescent rats. Performing research involving dual placement surgery in adolescent rats has concerns including: (1) similar aged rats will have marked differences in brain structure arrangements (failure to get consistent placement); (2) increased mortality rates and neurological events (seizures) since the cannulas are within a developing and expanding brain; (3) reduced mobility (adolescent rats tend to keep their head lower in response to the weight of the head cap), and (4) justified rejection of approval of experiments by review agencies (Drzewiecki and Juraska, 2020).

The current data replicate the findings reported by multiple laboratories that ABAE enhances EtOH consumption during adulthood (Figures 2, 3, and 6; McKinzie et al., 1996; Rodd-Henricks et al., 2002a, b; Schramm-Sapyta et al., 2008; Toalston et al., 2015; Amodeo et al., 2017, 2018; Spear, 2018; Rodd et al., 2020). The current findings replicate a previous report that pretreatment with an α7 NAM can prevent the ABAE-induced enhancement of adult EtOH consumption (Rodd et al., 2020; Figures 3 and 4). Thus, repeated datasets have now indicated that pretreatment with an α7 NAM can prophylactically prevent ABAE-induced enhancement of adult EtOH consumption presumably through regulation of the α7 receptor during ABAE exposure. Conversely, the data also indicates that modulation of the α7 receptor during ABAE can have bi-directional effects. Co-administration of a α7 NAM blocks ABAE-induced enhancements of adult alcohol consumption, while co-administration of a α7 PAM promotes the deleterious consequences of ABAE.

SB-277011-A is a D_3_ receptor antagonist and an α7 NAM (Zheng et al., 2016). SB has been tested as a potential pharmacotherapeutic for cocaine, nicotine, morphine, and alcohol. SB significantly decreases binge-like consumption of EtOH in C57B6 mice (Rice et al., 2015). Nicotine-seeking and self-administration can be inhibited by SB (Sabioni et al., 2016). Similarly, SB reduces cocaine and morphine self-administration and seeking (Rice et al., 2013). The SB compound reduces EtOH consumption in alcohol-preferring (P) rats (Thanos et al., 2005). Despite the extensive use of SB for the D_3_ receptor antagonism, there is no evidence reported that a D_3_ antagonist administered during adolescence has persistent effects inhibitory on behaviors during adulthood. The possibility that the effects of the SB compound on EtOH drinking in reported studies could be mediated in part by the α7 NAM actions of SB was not considered.

There are both endogenous α7 PAMs and a NAM. Secreted lymphocyte antigen-6/urokinase-type plasminogen activator receptor-related peptides (SLURP-1 and SLURP-2) are endogenous α7 PAMs that are expressed in all organs of the mammalian body (Chimienti et al., 2003). Kynurenic acid (KYNA) is an endogenous α7 NAM (KYNA active at glycine and ionotropic glutamate receptors; Kiss et al., 2003). Enhancing KYNA levels in the brain prevents nicotine-induced increases in dopamine (Erhardt et al., 2001) and glutamate (Konradsson-Geuken et al., 2009), reduces the reinforcing properties of Tetrahydrocannabinol (THC) (Justinova et al., 2013), and reduces nicotine craving and relapse (Secci et al., 2017). Although α7 PAMs have been reported to reduce the reinforcing properties of nicotine (Jackson et al., 2019), complete dose-response analyses have indicated that α7 PAMs produce leftward-shifts (increase sensitivity) in the dose-response curve for nicotine reward behaviors (Perkins et al., 2018). Recent clinical data have indicated that treatment with an α7 PAM increased nicotine craving and nicotine use (Perkins et al., 2018). Activation of the KYNA pathway reduces EtOH consumption and EtOH-induced dopamine release in the Acb (Giménez-Gómez et al., 2018).

A NAM does not reduce the activity of a neuron. Instead, NAMs prevent the increase or decrease activity of a receptor produced by ligands (Lopes et al., 2007). NAMs bind at allosteric sites on a receptor which are different from the binding sites for agonists and antagonists. For example, KYNA prevented choline-induced alterations of hippocampus neurons but did not affect the basal activity of these neurons (Smelt et al., 2018). The ability of α7 NAMs to prevent ligand alterations in receptor activity, without affecting basal activity, of neurons was replicate in six other α7 NAMs (Pocivavsek et al., 2014). Drugs that inhibit the α7 receptor during adolescence are associated with learning deficits during adulthood. Since α7 NAMs do not inhibit the basal activity of neurons containing the α7 receptor, there is no evidence that α7 NAMs administered during adolescence have this effect (Smelt et al., 2018). KYNA prenatally administered impairs adult learning, primarily through the glutamatergic and glycine actions of KYNA. However, KYNA administered during adolescence does not affect adult learning (Pocivavsek et al., 2014). Although the window of ABAE is fully unexplored, one would be tempted to find parallelism with effects of early-life exposure to environmental toxicants on the development of neurological disorders later in life *via* the LEARn pathway as well as across generations (*t*-LEARn; Lahiri et al., 2009, 2016).

Recent critical reviews of clinical interventions to treat adolescent problematic alcohol consumption have indicated no or small benefits of the treatments (Foxcroft and Tsertsvadze, 2011; Carney et al., 2016). Preclinical adolescent alcohol research should identify functional targets that could lead to the development of pharmacotherapeutics to counter the deleterious consequences of ABAE. The current data adds to the growing literature that cholinergic agents (in particular α7 NAMs) may be viable targets to treat ABAE and more extensive research with the goal of developing efficacious treatment is justified. Prevention is no longer a speculative concept. The findings reported here and in (Rodd et al., 2020) joined with the findings that galantamine blocked ABAE-induced increases in the expression of genes associated with the innate immune system (*TLR4* and *pNF-κB*) and histones/chromatin related genes (*RGE* and *HMGB1*) and during adulthood (Crews et al., 2021) to highlight replicated findings of preventative interventions against the persistent effects of ABAE.

Prevention has numerous benefits (prevention of harm/reduction of damage) compared to attempting to “reconstruct” a brain in a person who has suffered the etiological process to establish the AUD disorder. It is possible that novel targets will be more efficacious than α7 NAMs at preventing ABAE-induced neuroadaptations that persist throughout adulthood (or a combination treatment that includes α7 NAMs and other agents.

The development of preventative pharmacological treatment of other neuropsychological disorders is rapidly occurring. Prophylactic administration of ketamine (and ketamine metabolites) has been shown to block/prevent the development of fear-associated memories and post-traumatic stress disorder-like behaviors (PTSD; Henter et al., 2021; Zoladz et al., 2022). In addition, ketamine administered hours after exposure to the PTSD-inducing stimuli can similarly block/prevent the development of PTSD-like behaviors (Sala et al., 2022). Research attempting to develop efficacious treatments for Alzheimer’s Disease (AD) has produced very limited results. In contrast, a serendipitous finding may change the trajectory of AD in societies. Briefly, Cheng and colleagues performed a predictive functional structural analysis of FDA approved compounds that could interact at AD target sites and a lead target compound was determined to be sildenafil (Viagra; Fang et al., 2021). Examining over 7 million users of sildenafil, the researchers report that the common “lifestyle” cGMP-specific phosphodiesterase type 5 (PDE5) inhibitor reduced the likelihood of individuals to develop symptoms of AD in a 6-year period by 69% after controlling for a plethora of factors (Fang et al., 2021). Preventing neuropsychological disorders such as PTSD, AD, AUD, and addiction has such an overwhelming benefit to society that efforts should be focused on developing these treatments. Another possibility is that individuals will benefit from a combined approach in which prophylactic treatment against ABAE is used in conjunction with reversal treatments during adulthood (if needed). Gabapentin can normalize the alterations in sleep parameters induced by ABAE (Ehlers et al., 2018a, b), reverse ABAE-induced increases of glutamatergic activity in the hippocampus (Swartzwelder et al., 2017), and reverse ABAE-induced reductions of astrocyte-synaptic proximity (Healey et al., 2020). Donepezil can reverse other ABAE-induced alterations in the hippocampus (reduced dendritic spine density and expression of the *Fmr1*, Mulholland et al., 2018). The a7 NAChR system mediates many actions of Donepezil including neuroprotection, regulation of neuroimmune system, modulation of genetic expression (Takada-Takatori et al., 2008; Russo et al., 2017). These data suggest that the a7 NAChR system may also have utility to reverse the effects of ABAE during adulthood.

The effects of ABAE on adult behaviors are not limited to adult alcohol consumption. ABAE increases the risk for psychological disorders (depression, anxiety disorders, and schizophrenia), neurodegenerative disorders (AD, PD, and other dementia-related illnesses), and auto-immune diseases during adulthood (Harwood et al., 2010; Langballe et al., 2015; Schwarzinger et al., 2018; Coleman et al., 2019; Barnett et al., 2022; Tucker et al., 2022). ABAE also affects the reproductive system (hypogonadism and reduced sperm viability (Duca et al., 2019). The α7 receptor is expressed in the testes and on sperm cells. Cellular loss in the testes (like that observed following ABAE) is thought to occur through activation of the α7 receptor, and α7 receptors mediate sperm viability (Duca et al., 2019). Individuals who experience ABAE but do not develop AUD may still have these other, deleterious effects of ABAE. Therefore, any neuroprotection due to treatment with an α7 NAM could prevent such dysregulation in the adult α7 receptor system produced by excessive alcohol consumption, which would increase the risk of these disorders.

Nevertheless, the present work is the significant first step as it might cover several studies that suggest the effects of early-life exposures to chemicals and environmental and psycho-social factors on the later-life development of cognitive disorders, including AD (Maloney and Lahiri, 2016). Notably, the initial perturbation is maintained and later triggered via epigenetic mechanisms as conceptualized in the LEARn pathway, with evidence from animal studies and a small cohort of human autopsied brain tissue samples (Maloney and Lahiri, 2016). The present studies add to the epigenetic role on the effects of ABAE as described herein. For example, intermittent adolescent ethanol caused a persistent increase in adult H3K9me2, close to the NTRK1 gene and DNA methylation in ChAT promoter regions.

A novel α7 NAM has been created, BNC210. This compound has been approved for testing in human patients and has been shown to have little side-effects (Wise et al., 2020). BNC210 has been shown to reduce the expression of Seasonal Affect Disorder (SAD) and the altered neurocircuit activity profile associated with this disorder (Wise et al., 2020). BNC210 has also been shown to be efficacious at reducing the symptoms of General Anxiety Disorder (GAxD) and may have anxiolytic properties (Perkins et al., 2021). BNC210 has been given FastTrack Designation by the US FDA for the treatment of SAD and GAxD (Perkins et al., 2021). BNC210 is currently being assessed for treatment of PTSD. It is possible that BNC210 may have a similar prophylactic effect on preventing the development of PTSD as ketamine (another α7 NAM). Testing BNC210 on addictive behaviors could greatly advance the development of pharmacotherpeutics.

Overall, independent laboratories have reported that pretreatment with cholinergic agents (α7 NAMs and cholinesterase inhibitor) can preventatively block persistent effects of ABAE (Rodd et al., 2020; Crews et al., 2021). To briefly review, agonism of the α7 receptor synergistically activates posterior VTA DA neurons with EtOH (Figure 2), potentiating the actions of EtOH on the α7 receptor (PAM) during subthreshold exposure to ABAE that can produce enhanced intake of alcohol during adulthood (Figure 6), and ABAE induces persistent upregulation of the expression of *Chrna7* in the posterior VTA and AcbSh (Hauser et al., 2019a, 2021b) which is associated with an increase in protein expression of the α7 receptor in the posterior VTA (Waeiss et al., 2020), agonism of the α7 receptor results in a downregulation of ChAT (similar to ABAE; Dineley et al., 2015), and manipulations of the α7 receptor prevents the persistent effects of ABAE. Future studies need to determine the effects of pretreatment with α7 NAMs on the biological effect of exposure to ABAE in the adolescent brain and body. Currently, the actual biological effects of ABAE in adolescents are understudied. Extensive research needs to be conducted (and independently replicated) to advance the use of α7 NAMs as a preventative treatment against ABAE.

## Data availability statement

All relevant data is contained within the article: The original contributions presented in the study are included in the article, further inquiries can be directed to the corresponding author.

## Ethics statement

The animal study was reviewed and approved by Animals care facilities at IUSM are fully accredited by the Association for the Assessment and Accreditation of Laboratory Animal Care. Research performed in the current experiments were approved by the IUSM Institutional Animal Care and Use Committee (IUSM IACUC) and were in accordance with the guidelines of the Institutional Care and Use Committee of the National Institute on Drug Abuse, the NIH, and the Guide for the Care and Use of Laboratory Animals (2011).

## Author contributions

The experiments were conceived by ZR, DL, SH, HS, and RB. The microdialysis experiment was performed by RW, SS, EE, SH, and ZR. The EtOH drinking experiment was performed by RW, SS, SH, RB, WT, and ZR. HPLC analyses were performed by EE, RW, and SH. Statistical analyses were performed by RB, DL, SS, and ZR. The manuscript was written by ZR, SH, DL, and RB. All authors contributed to the article and approved the submitted version.
